# The Shape-Shifting Myeloma: Adaptive Plasticity as a Hallmark of Relapse and Refractoriness

**DOI:** 10.3390/cancers18121873

**Published:** 2026-06-08

**Authors:** Maria Elisa Nasso, Adele Bottaro, Demetrio Gerace, Sabina Russo, Donato Mannina, Alessandro Allegra

**Affiliations:** 1 Hematology Unit, Department of Human Pathology in Adulthood and Childhood “Gaetano Barresi”, University of Messina, Via Consolare Valeria, 98125 Messina, Italy; mariaelisanasso@gmail.com (M.E.N.); adelebottarp15@gmail.com (A.B.); demetriogabriele.gerace@polime.it (D.G.); sabina.russo@polime.it (S.R.); 2 Hematology Unit, Oncology-Hematology Department, Azienda Ospedaliera Papardo, 98158 Messina, Italy; donamanni@gmail.com

**Keywords:** multiple myeloma, relapsed/refractory disease, tumor plasticity, cell state switching, clonal evolution, microenvironment, epigenetics, immunotherapy resistance

## Abstract

Relapsed and refractory multiple myeloma remains the principal cause of myeloma-related mortality despite major advances in therapeutic options, including proteasome inhibitors, immunomodulatory drugs, monoclonal antibodies, and T-cell-based immunotherapies. Conventional resistance models based on linear genetic clonal evolution inadequately explain reversible drug resistance, heterogeneous responses, and relapse after deep remissions. Emerging data from longitudinal genomics, single-cell analyses, and functional studies support a paradigm in which relapsed and refractory multiple myeloma is driven by adaptive plasticity rather than irreversible genetic change alone. Myeloma cells undergo reversible cell state transitions through transcriptional, epigenetic, metabolic, and proteostatic reprogramming, shaped by bone marrow microenvironmental cues and immune pressure. This narrative review integrates current evidence supporting adaptive plasticity as a central driver of therapeutic failure in relapsed and refractory multiple myeloma and discusses clinical and translational implications, highlighting adaptive treatment strategies and approaches targeting phenotypic flexibility to improve durability of response.

## Simple Summary

Despite major therapeutic advances, multiple myeloma remains largely incurable, with most patients eventually developing relapsed and refractory disease. Traditional explanations for treatment failure have primarily focused on genetic clonal evolution and the selection of resistant subclones. Increasing evidence, however, indicates that myeloma cells dynamically adapt to therapeutic pressure through reversible, non-genetic mechanisms. This review frames relapsed and refractory multiple myeloma as a disease of adaptive plasticity, driven by cell state switching, metabolic and proteostatic reprogramming, microenvironmental interactions, and immune evasion. Understanding these adaptive processes provides an integrated biological framework to explain therapeutic failure in the modern treatment era and supports the development of adaptive, state-aware therapeutic strategies aimed at achieving durable disease control.

## 1. Introduction

Multiple myeloma (MM) is a biologically and clinically heterogeneous plasma cell malignancy characterized by the progressive accumulation of clonal plasma cells within the bone marrow microenvironment, ultimately leading to organ damage and disease-related mortality [1]. Despite substantial therapeutic advances over the past two decades—including proteasome inhibitors, immunomodulatory drugs, monoclonal antibodies, and, more recently, T-cell-redirecting immunotherapies—MM remains largely incurable, with the majority of patients eventually developing relapsed and refractory disease (RRMM).

The clinical course of RRMM is marked by progressively shorter response durations and the emergence of resistance to therapies with distinct mechanisms of action, highlighting the remarkable adaptive capacity of malignant plasma cells [2]. Traditionally, relapse and refractoriness have been interpreted primarily within the framework of genetic clonal evolution, whereby therapeutic pressure selects for resistant subclones harboring advantageous genomic alterations [3,4].

However, while clonal evolution remains a foundational concept, this framework alone does not fully account for several key features of RRMM.

An emerging paradigm therefore conceptualizes RRMM as a disease driven not only by clonal selection but also by adaptive plasticity, defined as the capacity of myeloma cells to dynamically and reversibly modify their functional state in response to different stress [5,6]. This perspective provides a unifying explanation for heterogeneous treatment responses, minimal residual disease persistence, and post-remission relapse [7]. Importantly, therapeutic resistance may reflect context-dependent and potentially reversible phenotypic reprogramming rather than the inevitable dominance of genetically fixed resistant clones [8].

Framing RRMM as a dynamically adaptive system has important implications for understanding disease progression and for the development of therapeutic strategies. Specifically, it suggests that targeting the mechanisms underlying phenotypic flexibility may be as critical as targeting specific genetic lesions, with the goal of improving the durability of clinical responses [9].

**Figure 1:**
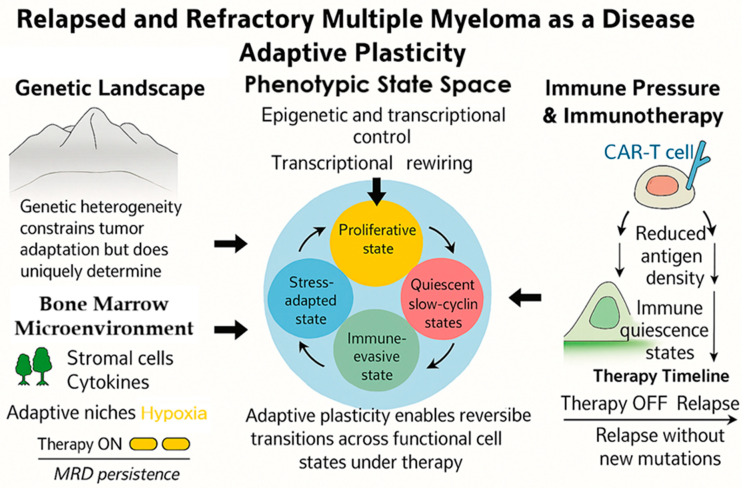
Genetic heterogeneity defines the boundaries of potential tumor behavior but does not uniquely determine therapeutic resistance. Adaptive plasticity enables myeloma cells to reversibly transition across functional phenotypic states in response to therapeutic pressure, bone marrow microenvironmental cues, metabolic stress, and immune attack. Epigenetic regulation orchestrates transcriptional rewiring, stabilizing survival-optimized states that support minimal residual disease persistence and relapse without the requirement for new dominant genetic alterations.

In this review, we synthesize current evidence supporting adaptive plasticity as a central driver of relapse and refractoriness in multiple myeloma. We discuss the molecular determinants of plasticity, including epigenetic regulation, metabolic rewiring, and microenvironmental crosstalk, as well as their impact on drug resistance and immune escape (Figure 1). Finally, we explore emerging therapeutic strategies aimed at constraining plasticity, enforcing differentiation, or exploiting adaptive vulnerabilities, with the ultimate goal of achieving more durable disease control in RRMM.

During manuscript preparation, Microsoft 365 Copilot (GPT-5 chat model) was used solely as an auxiliary tool for language refinement, figure conceptualization, and organizational support.

## 2. Clonal Evolution in Multiple Myeloma: Achievements and Limitations

Genomic and cytogenetic studies have firmly established clonal evolution as a defining feature of multiple myeloma, particularly in the context of disease progression and therapeutic resistance. From early precursor stages to relapsed disease, myeloma development follows a branching evolutionary pattern in which distinct subclones coexist, expand, or regress over time under selective pressures [10,11]. The application of next-generation sequencing has revealed substantial spatial and temporal heterogeneity, demonstrating that myeloma is rarely driven by a single dominant clone, but rather by a dynamic ecosystem of genetically diverse populations [12]. Longitudinal analyses further indicate that therapeutic exposure acts as a powerful evolutionary bottleneck, reshaping clonal architecture by selectively eliminating sensitive populations while permitting the survival and expansion of resistant ones [3].

Within this Darwinian framework, clonal evolution has successfully explained several core features of disease progression, including the enrichment of high-risk cytogenetic abnormalities, the accumulation of mutations in signaling pathways such as MAPK and DNA damage response networks, and the progressive increase in genomic complexity across successive lines of therapy [13]. In this sense, clonal evolution has provided an essential conceptual foundation for understanding why relapse is nearly inevitable in multiple myeloma.

However, despite its explanatory power, a purely genetic model increasingly appears insufficient to fully account for the clinical and biological behavior of RRMM. Notably, disease relapse may occur in the absence of newly acquired dominant driver mutations, indicating that genetic change is not invariably required for tumor re-expansion. Moreover, distinct relapses arising within the same patient may display divergent genetic features yet converge toward highly similar transcriptional, phenotypic, and functional states [14]. These observations suggest that non-genetic mechanisms play a substantial role in shaping therapeutic resistance.

A further limitation of clonal evolution lies in its inability to fully explain the rapid emergence of resistance observed in clinical settings. Resistance frequently develops on timescales that are difficult to reconcile with the accumulation and fixation of multiple advantageous mutations alone [3]. This is particularly evident in advanced RRMM, where resistance to therapies with distinct mechanisms of action can arise in quick succession, and where cross-resistance to structurally and mechanistically unrelated agents is common [15]. Such kinetics strongly suggest the presence of pre-existing adaptive capacities rather than exclusively de novo genetic innovation.

In addition, clonal models struggle to account for the reversibility observed in certain resistance phenotypes. Both experimental and clinical evidence indicate that myeloma cells can enter transient drug-tolerant states characterized by reduced proliferation, altered stress responses, and enhanced survival, and subsequently revert to more proliferative configurations upon relief of selective pressure [8]. These reversible transitions are difficult to reconcile with stable genetic alterations and instead point toward non-genetic adaptive processes [16].

Another critical limitation concerns the static nature of most genomic analyses. Bulk sequencing approaches provide a snapshot of tumor composition but may obscure the functional heterogeneity present at the single-cell level. Even when resistant subclones are identified, their detection does not fully explain how they persist, adapt, and ultimately dominate under dynamic therapeutic conditions [17]. In this regard, genetic alterations define the potential space of adaptation but do not fully determine the phenotypic trajectories that myeloma cells actually pursue.

Furthermore, clonal evolution frameworks often underappreciate the influence of the bone marrow microenvironment, which exerts profound effects on selective pressures and adaptive outcomes [18]. Identical genetic clones can display markedly different behaviors depending on microenvironmental context, including interactions with stromal cells, immune populations, and local metabolic conditions. This context dependency limits the predictive capacity of genetics alone and suggests that selection operates more directly on phenotypes rather than genotypes [19].

Taken together, these considerations indicate that clonal evolution, while necessary, is not sufficient to fully explain the biology of RRMM. Genetic heterogeneity provides the substrate upon which adaptation occurs, but does not capture the mechanisms that enable myeloma cells to rapidly and repeatedly adjust their functional state in response to stress. Resistance emerges not solely from the selection of more fit clones, but from the capacity of malignant plasma cells to explore and occupy alternative phenotypic states compatible with survival [15,16,20].

Recognizing these limitations does not diminish the importance of clonal evolution; rather, it reframes it as one component of a broader adaptive system. Genetic changes shape the landscape of possible states, whereas adaptive plasticity determines how myeloma cells navigate this landscape over time, particularly under sustained therapeutic pressure in RRMM [17]. In this context, integrating clonal genetics with non-genetic adaptive mechanisms is essential to achieve a more comprehensive and clinically relevant model of disease progression, which will be further explored in the following sections.

## 3. Conceptualizing Adaptive Plasticity in Relapsed and Refractory Myeloma

Tumor plasticity refers to the reversible capacity of malignant cells to transition between distinct functional states in response to environmental or therapeutic stress. In the context of RRMM, adaptive plasticity represents a fundamental biological property that enables myeloma cells to survive and persist despite sustained selective pressure. Unlike clonal evolution, which relies on the fixation of heritable genetic alterations, adaptive plasticity operates through dynamic and largely non-genetic reprogramming, allowing rapid phenotypic adjustment without permanent changes to the genomic sequence [21,22].

Within this framework, resistance emerges as a flexible, context-dependent phenotype. Myeloma cells can dynamically modify transcriptional programs, epigenetic states, and signaling pathways in response to stress, thereby expanding the range of accessible functional states. This conceptual shift reframes RRMM as a system in which cell behavior is continuously reshaped by internal and external constraints rather than fixed by underlying genomic alterations alone [23,24,25].

### 3.1. Biological and Phenotypic Features of Adaptive Plasticity

A defining feature of adaptive plasticity is reversibility. Myeloma cells can enter drug-tolerant configurations characterized by reduced proliferation, altered transcriptional programs, and enhanced reliance on survival pathways, and subsequently revert to more proliferative states upon relief of selective pressure [23]. These transitions are not deterministic but often stochastic, enabling phenotypic heterogeneity even within genetically homogeneous populations.

Adaptive plasticity also operates across multiple biological scales. At the molecular level, it involves transcriptional rewiring, epigenetic remodeling, and dynamic signaling adaptations. At the cellular level, it manifests as changes in proliferation, metabolism, and differentiation. At the tissue level, it is influenced by spatial heterogeneity within the bone marrow, where distinct niches impose different constraints and selectively stabilize specific cell states [26,27,28].

An important consequence of this multilevel organization is that genetically distinct clones can converge toward similar phenotypic states. Conversely, cells sharing the same genetic background may display markedly different functional behaviors depending on environmental context. In this sense, genetic determinants define the boundaries of possible behaviors, whereas plasticity governs how these possibilities are explored in real time [29].

### 3.2. Clinical Relevance of Adaptive Plasticity

Adaptive plasticity provides a coherent explanation for several clinical features of RRMM that are insufficiently accounted for by genetic models alone. These include the persistence of measurable residual disease (MRD), the transient nature of many resistance states, and the recurrence of disease following treatment discontinuation or modification.

Residual disease populations are often phenotypically distinct from the dominant tumor at diagnosis, displaying quiescent, stem-like, or stress-adapted characteristics that enable long-term survival under adverse conditions. Importantly, these cells are not necessarily genetically unique but instead represent specific functional states stabilized by epigenetic and microenvironmental signals [24]. Their persistence reflects the ability of myeloma cells to occupy survival-optimized configurations that are not effectively targeted by conventional therapies.

More broadly, adaptive plasticity reframes relapse as the re-expansion of permissive phenotypic states rather than the emergence of new genetically defined clones. Disease progression thus reflects dynamic shifts in functional state distributions within the tumor ecosystem [22].

### 3.3. Therapeutic Implications

Recognizing adaptive plasticity as a central driver of RRMM has important implications for therapeutic strategies. If resistance is mediated by reversible and context-dependent phenotypic states, then treatment failure does not necessarily indicate permanent insensitivity but may reflect the induction of transient survival programs. This perspective supports approaches that go beyond simple clonal targeting and instead aim to interfere with the mechanisms enabling phenotypic flexibility. Potential strategies include constraining epigenetic variability, disrupting stress-adaptive signaling pathways, modulating microenvironmental inputs, or enforcing terminal differentiation states. By limiting the accessible “state space” available to myeloma cells, such interventions may reduce adaptive capacity and improve the durability of therapeutic responses [25,30].

Importantly, adaptive plasticity also implies that therapeutic sequencing and timing become critical variables. Treatments may reshape the phenotypic landscape in ways that influence subsequent responses, suggesting that adaptive or state-aware treatment strategies could be more effective than static approaches [25].

Taken together, adaptive plasticity provides an integrated framework that links genetic heterogeneity, non-genetic reprogramming, and microenvironmental influence into a unified model of RRMM biology. Within this model, genetics defines the landscape of possible states, while plasticity determines how myeloma cells navigate that landscape over time.

Understanding RRMM as a dynamically adaptive system rather than a static collection of resistant clones is essential for interpreting disease progression and for designing next-generation therapeutic strategies. This conceptual foundation underpins the subsequent sections of this review, which explore the molecular, cellular, and clinical dimensions of adaptive plasticity in greater detail.

## 4. Cell State Switching as a Driver of Relapsed and Refractory Disease

Cell state switching represents a core functional manifestation of adaptive plasticity in RRMM, describing the ability of malignant plasma cells to dynamically and reversibly transition between distinct phenotypic configurations. These transitions occur not only in response to therapeutic pressure but also as a consequence of microenvironmental cues and intrinsic regulatory programs, collectively shaping tumor behavior over time. Unlike fixed subclonal architectures, cell states are fluid and interconvertible, enabling myeloma cells to rapidly adapt to changing selective conditions [31].

Within this framework, RRMM is characterized not simply by the selection of resistant clones but by the continuous redistribution of cells across functional states, each associated with specific survival advantages under defined contexts. This dynamic equilibrium between states underpins both treatment resistance and disease recurrence.

### 4.1. Mechanistic Basis of Cell State Transitions

Cell state switching is driven by coordinated and reversible reprogramming across multiple regulatory layers. At the transcriptional level, therapeutic and microenvironmental stress induces activation of alternative gene expression programs that shift cellular priorities from proliferation toward survival. These changes are often mediated by stress-responsive transcription factors and signaling pathways, which rapidly rewire cellular outputs without requiring genetic alterations [31].

Epigenetic regulation plays a central role in stabilizing these transient states. Chromatin remodeling, histone modifications, and DNA methylation dynamically alter accessibility of regulatory regions, allowing cells to enter and maintain drug-tolerant configurations. Importantly, these epigenetic changes are reversible, enabling bidirectional transitions between proliferative and stress-adapted states, and providing a mechanistic basis for the plasticity observed in RRMM [20,32].

Metabolic reprogramming further reinforces state transitions by supporting the energetic and biosynthetic demands of survival-oriented phenotypes. For example, shifts toward mitochondrial oxidative metabolism and enhanced redox control enable myeloma cells to persist under nutrient limitation, oxidative stress, and therapeutic exposure. These metabolic adaptations are tightly integrated with transcriptional and epigenetic programs, forming a self-consistent adaptive state [33,34,35,36,37].

In addition, the bone marrow microenvironment actively instructs and stabilizes specific cell states. Signals derived from stromal cells, extracellular matrix interactions, cytokines, and hypoxic niches activate survival pathways and reinforce non-proliferative or immune-evasive phenotypes. Identical genetic clones may therefore occupy distinct functional states depending on their spatial and microenvironmental context [38,39].

### 4.2. Functional States Associated with RRMM

Under these regulatory influences, myeloma cells can adopt multiple interconvertible states with distinct functional properties. One of the most well-characterized is a quiescent or slow-cycling state, in which reduced proliferation confers resistance to therapies targeting dividing cells, while preserving long-term clonogenic potential [32].

Another major configuration is a stress-adapted state characterized by rewiring of proteostasis and survival signaling pathways. In this state, myeloma cells reduce dependence on proteasomal degradation, increase autophagic flux, and enhance stress-response pathways, thereby tolerating prolonged exposure to proteasome inhibitors and other cytotoxic agents [33,34].

Cell state switching may also involve partial reprogramming of differentiation status, with transient downregulation of plasma cell-associated transcriptional programs and activation of alternative regulatory networks. These shifts reduce dependency on lineage-specific vulnerabilities and broaden the phenotypic space accessible to malignant cells under selective pressure [35].

Importantly, these states are not static or mutually exclusive. Within the same tumor population, cells continuously transition between configurations, generating dynamic heterogeneity that ensures survival under diverse and changing conditions [36,37].

### 4.3. Reversibility and Resistance

A defining feature of cell state switching in RRMM is reversibility. Cells that enter quiescent or stress-adapted states during therapy can re-enter proliferative programs upon treatment withdrawal or modification, driving disease re-expansion. This reversible behavior explains the persistence of minimal residual disease and the frequent recurrence of clinically detectable tumors following apparently effective treatment [20,32].

Rather than representing permanently resistant subclones, these cells constitute a reservoir of adaptable phenotypes capable of reactivating tumor growth. Resistance is therefore better understood as a transient and context-dependent property emerging from state occupancy rather than fixed genetic change.

### 4.4. Therapeutic Vulnerabilities Emerging from Cell State Plasticity

Recognizing cell state switching as a driver of RRMM reveals specific and actionable therapeutic vulnerabilities. Targeting epigenetic regulators that stabilize adaptive states may prevent entry into or maintenance of drug-tolerant configurations. Similarly, disruption of metabolic dependencies—such as mitochondrial function or redox balance—may selectively impair stress-adapted cells that rely on these pathways for survival [34].

Interfering with proteostasis networks, particularly autophagy and lysosomal pathways, represents another strategy to exploit vulnerabilities of cells that have shifted away from proteasome dependence. In addition, targeting microenvironmental interactions, including adhesion-mediated signaling and cytokine networks, may destabilize protective niches that sustain adaptive states [38].

Importantly, the dynamic nature of cell state transitions suggests that therapeutic timing and sequencing are critical. Strategies that combine cytotoxic agents with interventions that prevent or reverse entry into tolerant states may limit the emergence of functional resistance. Adaptive or state-aware treatment approaches may therefore be more effective than static regimens in controlling RRMM [40].

In summary, cell state switching provides the mechanistic foundation of adaptive plasticity in RRMM, linking molecular reprogramming to clinical resistance and relapse. Through reversible transitions across multiple functional states, myeloma cells achieve a level of adaptability that cannot be explained by genetic evolution alone.

Understanding these processes at a mechanistic level not only clarifies the biology of RRMM but also identifies novel opportunities for therapeutic intervention aimed at constraining phenotypic flexibility and improving the durability of clinical responses.

**Figure 2:**
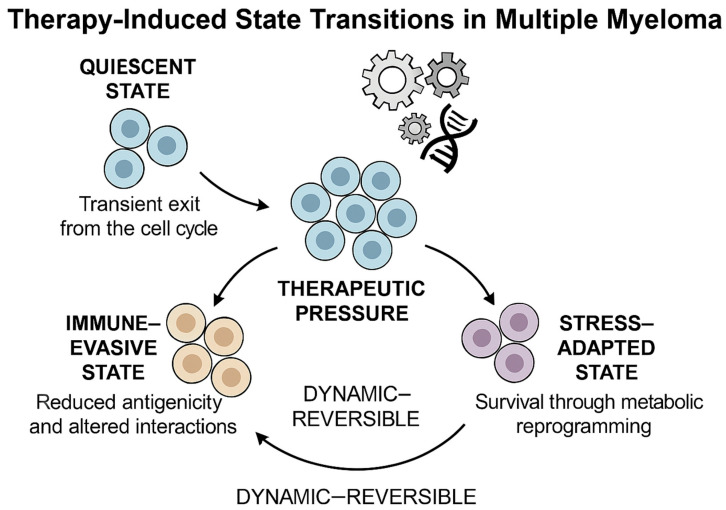
Therapeutic stress promotes dynamic transitions between quiescent, stress-adapted, and immune-evasive states through transcriptional, epigenetic, and metabolic reprogramming, enabling persistence and relapse without fixed genetic resistance.

These reversible transitions ensure survival under sustained therapeutic stress and provide a reservoir for disease re-expansion upon treatment modulation [Figure 2].

## 5. Metabolic and Proteostatic Adaptation in Relapsed and Refractory Myeloma

Metabolic and proteostatic adaptation represent critical functional axes through which multiple myeloma cells sustain survival under therapeutic and microenvironmental stress. As professional immunoglobulin-producing cells, plasma cells inherently operate near the limits of proteostasis, rendering myeloma particularly dependent on finely tuned protein quality control and bioenergetic systems. In RRMM, adaptive remodeling of these systems enables malignant cells to tolerate sustained pharmacologic pressure and contributes significantly to the development of drug refractoriness [41,42].

A defining feature of advanced myeloma is metabolic flexibility. Under therapeutic stress, myeloma cells dynamically rewire their metabolic programs to optimize energy production, redox balance, and biosynthetic capacity [43,44]. While newly diagnosed disease is often characterized by high glycolytic flux, RRMM cells frequently shift toward increased mitochondrial oxidative capacity, enhancing fitness under stress conditions [45,46]. This metabolic shift promotes survival under adverse conditions and facilitates transition toward stress-adapted, therapy-tolerant cellular states, while also contributing to resistance to apoptosis through modulation of mitochondrial dynamics and redox balance [46,47,48].

In parallel, RRMM is marked by adaptive reprogramming of proteostatic control. Proteasome inhibition induces compensatory pathways, including autophagy and lysosomal degradation, which restore proteome balance and reduce proteotoxic stress [49,50]. These mechanisms stabilize drug-tolerant configurations by shifting cellular dependency away from the proteasome toward alternative survival pathways. Autophagy plays a particularly prominent role in this context by facilitating the degradation of misfolded proteins and damaged organelles, thereby sustaining survival during therapeutic stress [51,52]. Importantly, metabolic and proteostatic adaptations are tightly interconnected, forming a self-reinforcing adaptive loop that stabilizes stress-tolerant states [46,53,54].

**Figure 3:**
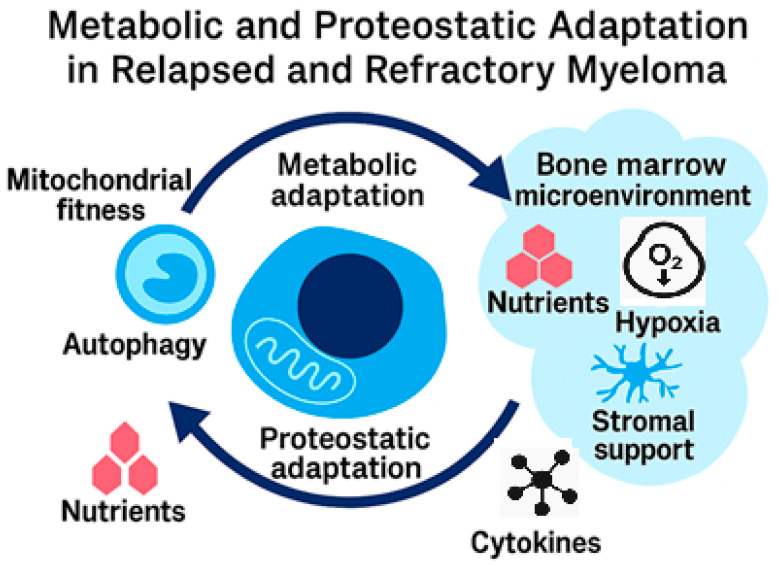
Multiple myeloma cells adapt to therapeutic stress through coordinated metabolic and proteostatic remodeling. Enhanced mitochondrial fitness sustains energy production, redox balance, and resistance to mitochondrial apoptosis. Autophagy compensates for proteasome inhibition and supports mitochondrial metabolism, contributing to proteostatic adaptation. These processes form a self-reinforcing adaptive loop that promotes cell survival. Bone marrow microenvironmental cues, including nutrients, cytokines, hypoxia, and stromal support, actively induce and stabilize these stress-tolerant cellular states, facilitating drug refractoriness and disease relapse.

From a clinical perspective, these processes explain the persistence of viable myeloma cells during prolonged therapy and their ability to re-expand upon treatment modification or withdrawal [44,55]. These adaptations also reveal actionable therapeutic vulnerabilities, including dependence on mitochondrial function, redox homeostasis, and compensatory proteostatic pathways, suggesting potential sensitivity to mitochondrial inhibitors, oxidative stress-inducing strategies, or autophagy-targeting approaches [56] (Table 1 and Figure 3). Taken together, metabolic and proteostatic adaptation sustain adaptive plasticity by stabilizing stress-tolerant cellular states that enable persistence under therapeutic pressure and contribute to relapse in RRMM.

## 6. Microenvironment-Driven Plasticity and Adaptive Niches

Bone marrow microenvironment actively drives adaptive plasticity by inducing and stabilizing distinct cellular states associated with drug tolerance in RRMM. Microenvironmental signals continuously instruct malignant plasma cells to adopt survival-optimized functional states, generating protective niches that promote drug tolerance, facilitate immune evasion, and ultimately support disease persistence [57].

Adhesion-mediated interactions with stromal cells and extracellular matrix components activate intracellular survival pathways that reduce sensitivity to apoptosis and therapeutic agents [58,59,60]. These interactions promote transitions toward microenvironment-dependent drug-tolerant states that remain reversible upon disruption of niche-derived signals. In parallel, cytokines and growth factors such as IL-6, IGF-1, and SDF-1 further reinforce adaptive states by modulating signaling networks and differentiation trajectories [61,62,63,64,65,66]. Rather than inducing uniform responses, these signals contribute to phenotypic diversification, increasing the likelihood that subsets of cells will survive therapeutic pressure.

Hypoxia represents another major driver of plasticity within the bone marrow microenvironment. Oxygen gradients induce metabolic and transcriptional reprogramming that supports survival in nutrient-limited and poorly perfused niches [67,68,69,70]. These conditions selectively stabilize stress-adapted phenotypes and contribute to the spatial segregation of resistant cellular populations. In addition, the immune microenvironment promotes adaptive plasticity by shaping immune-evasive states, through induction of dysfunction in effector cells and expansion of immunosuppressive populations [71,72,73].

Collectively, these mechanisms demonstrate that the microenvironment does not merely act as a passive sanctuary but actively shapes phenotypic states that sustain tumor survival. Targeting adhesion-mediated signaling, cytokine networks, or hypoxia-driven pathways may therefore destabilize these protective niches and enhance therapeutic efficacy [74,75,76,77,78,79]. A central principle emerging from this section is that microenvironmental cues continuously regulate and stabilize adaptive states, thereby playing a critical role in reversible drug resistance and disease recurrence in RRMM.

## 7. Adaptive Plasticity and Resistance to Immunotherapy

Adaptive plasticity enables dynamic immune evasion through reversible state transitions, thereby limiting the durability of immunotherapy responses in RRMM. Although immunotherapeutic strategies have transformed the treatment landscape, resistance remains common and is increasingly recognized as being mediated by non-genetic, reversible mechanisms [80,81].

A central mechanism of immune escape is the dynamic modulation of target antigen expression. Rather than permanent loss, myeloma cells frequently downregulate surface antigens such as BCMA or CD38 under immune pressure and subsequently re-express them once selective pressure is relieved [82,83,84,85,86]. This process reflects transitions into immune-evasive states rather than fixed genetic resistance.

Adaptive plasticity also enables broader immune-evasive phenotypes characterized by altered signaling, reduced interferon responsiveness, and activation of stress-adaptive transcriptional programs [87,88]. In parallel, cell state switching toward quiescent or metabolically reprogrammed states reduces susceptibility to immune-mediated killing, as these states are less compatible with efficient immune recognition and cytotoxicity [89,90,91,92,93].

Importantly, resistance to different classes of immunotherapy often converges on similar phenotypic endpoints despite mechanistic diversity, supporting the concept that immune resistance is primarily a plastic and reversible process. From a therapeutic perspective, this suggests that strategies aimed at stabilizing antigen expression, restoring immune sensitivity, or preventing transition into immune-evasive states may enhance the durability of immune responses [94,95,96,97]. Taken together, immune resistance in RRMM reflects dynamic adaptation, in which tumor cells transiently occupy immune-evasive states that can potentially be therapeutically targeted.

Finally, longitudinal monitoring of phenotypic and transcriptional adaptations may be more informative than static genomic assessments in predicting and managing immunotherapy resistance [90,98,99].

## 8. Epigenetic Regulators of Immune Plasticity in Relapsed and Refractory Myeloma

Epigenetic processes dynamically regulate transcriptional programs that determine immune visibility, lineage identity, and stress responsiveness without requiring permanent genomic alterations [100,101,102].

Through mechanisms such as DNA methylation, histone modification, and chromatin remodeling, myeloma cells can reversibly modulate the expression of immune-relevant genes, including tumor antigens and components of antigen presentation pathways [103,104,105,106]. These reversible changes underpin transitions between immune-sensitive and immune-evasive states, enabling tumor cells to adapt rapidly to immunological pressure.

Epigenetic plasticity also plays a key role in resistance to CAR-T cells and bispecific antibodies by regulating pathways involved in immune synapse formation, apoptotic signaling, and cellular stress responses [107,108,109,110]. These processes allow tumor cells to attenuate immune-mediated killing while maintaining the capacity to revert to more immunogenic states under different conditions [111,112].

Importantly, epigenetic regulation is closely integrated with microenvironmental signals, including cytokines and hypoxia, which further shape chromatin states and transcriptional outputs [113,114]. From a therapeutic perspective, this plasticity suggests that epigenetic therapies may restore immune sensitivity by reprogramming resistant phenotypes rather than directly inducing cytotoxicity [115,116,117,118]. A central principle is that epigenetic regulation enables rapid and reversible modulation of immune-relevant phenotypes, providing a mechanistic basis for adaptive immune resistance in RRMM.

## 9. Clinical and Translational Implications of Adaptive Plasticity

This section integrates the concept of adaptive plasticity into a clinical framework, emphasizing its implications for resistance, treatment design, and therapeutic strategies in RRMM. Reconceptualizing RRMM as a disease driven by adaptive plasticity shifts the understanding of resistance from a fixed genomic endpoint to a dynamic and reversible process [119,120,121].

In this context, treatment failure often reflects the induction or selection of survival-optimized phenotypic states rather than permanent drug resistance [59,122,123]. Sequential therapeutic strategies based solely on non-overlapping mechanisms of action may inadvertently promote cross-resistance by favoring broadly tolerant cellular states capable of surviving diverse stresses [122,124,125,126]. This highlights how therapy-induced state transitions continuously reshape the phenotypic landscape and influence subsequent treatment responses.

Immunotherapy provides a clear example of this dynamic behavior, as adaptive modulation of antigen expression and immune sensitivity allows tumor cells to evade immune control despite initial responses [85,127,128,129]. These observations underscore the importance of considering phenotypic flexibility when designing therapeutic strategies.

From a translational perspective, adaptive plasticity supports the development of state-aware treatment approaches aimed not only at tumor eradication but also at limiting the capacity for phenotypic adaptation. This includes rational sequencing strategies, combination therapies targeting both dominant tumor populations and adaptive states, and interventions designed to constrain plasticity itself. Taken together, a plasticity-centered framework supports a shift from static treatment paradigms toward adaptive therapeutic strategies aimed at improving the durability of clinical responses in RRMM [130,131,132].

## 10. Conclusions 

Relapsed and refractory multiple myeloma represents the clinical manifestation of a malignancy capable of continuously adapting to therapeutic and environmental constraints. Rather than reflecting the inevitable outgrowth of fixed genetically resistant clones, disease persistence and recurrence appear to arise from the capacity of myeloma cells to dynamically reorganize their functional identity across treatment lines. This adaptive behavior operates through coordinated modulation of transcriptional programs, metabolic and proteostatic dependencies, immune interactions, and microenvironmental engagement [2,133,134].

Within this framework, therapeutic pressure acts not only as a selective force but also as a driver of phenotypic reconfiguration, reshaping the functional composition of the tumor without necessarily altering its genetic architecture. The resulting resilience explains why profound cytoreduction and even transient MRD negativity frequently fail to translate into durable disease control. Relapse therefore reflects re-expansion from permissive cellular states rather than true biological reinitiation of disease [135].

Recognizing RRMM as a dynamically adaptive system carries important implications for how therapeutic success is defined and pursued [20,136].

However, while an increasing body of evidence supports adaptive plasticity as a critical driver of relapse and refractoriness in multiple myeloma, it is important to acknowledge that several mechanistic insights—particularly those derived from high-resolution single-cell and multi-omics studies—remain emergent, albeit largely consistent with established biological and clinical observations. Moreover, adaptive plasticity should not be interpreted as a process dissociated from the underlying genomic architecture of the tumor. On the contrary, the genetic and cytogenetic background of myeloma cells defines the boundaries within which phenotypic adaptation can occur, with specific karyotypic and mutational contexts constraining, biasing, or canalizing accessible adaptive states. In this sense, plasticity does not supersede genomic determinants of disease behavior, nor does it operate as an independent or dominant axis of resistance. Rather, therapeutic resistance in RRMM arises from the dynamic interplay between a relatively stable genetic substrate and flexible, non-genetic reprogramming mechanisms that modulate functional behavior under selective pressure. Recognizing this hierarchical yet interactive relationship is essential to avoid overinterpretation of plasticity-driven models and to integrate them meaningfully with genomic frameworks in both translational research and clinical decision-making.

## Figures and Tables

**Table 1:** Multidimensional adaptive plasticity as a driver of relapse and refractoriness in multiple myeloma.

Dimension of Plasticity	Adaptive Mechanisms	Functional Impact in RRMM	Clinical and Translational Implications
Genetic background	Clonal heterogeneity; branching evolution; persistence of early clones without new dominant mutations	Defines the range of accessible adaptive states but does not uniquely determine resistance	Genetic profiling alone insufficient to predict relapse or refractoriness
Epigenetic regulation	Dynamic DNA methylation; histone modifications; chromatin remodeling; non-coding RNA networks	Stabilization of reversible drug-tolerant and immune-evasive phenotypes	Epigenetic targeting may constrain phenotypic flexibility and restore sensitivity
Cell state switching	Reversible transitions between proliferative, quiescent, stress-adapted, and immune-evasive states	Rapid, non-genetic tolerance to therapy; persistence of minimal residual disease	Necessitates targeting of non-proliferative and stress-tolerant cell states
Metabolic adaptation	Shift toward mitochondrial oxidative metabolism; enhanced mitochondrial fitness; redox optimization	Survival under hypoxia, nutrient limitation, and therapeutic stress	Reveals vulnerabilities to metabolic and mitochondrial targeting strategies
Proteostatic adaptation	Reduced proteasome dependence; induction of autophagy; enhanced lysosomal and chaperone activity	Resistance to proteasome inhibitors; attenuation of proteotoxic stress	Supports combination therapies targeting alternative protein quality control pathways
Metabolism–proteostasis crosstalk	Autophagy–mitochondria reciprocity; substrate recycling; redox coupling	Stabilization of stress-tolerant cellular configurations	Disruption of this adaptive loop may collapse survival states
Bone marrow microenvironment	Stromal interactions; cytokines; nutrient availability; hypoxia; spatially distinct niches	Induction and maintenance of adaptive, drug-tolerant states; sanctuary niches	Targeting tumor–microenvironment interactions essential for durable disease control
Immune plasticity	Reversible antigen modulation; immune-evasive transcriptional programs; immune synapse remodeling	Functional resistance to monoclonal antibodies, CAR-T cells, and bispecific antibodies	Immunotherapies should be combined with plasticity-constraining approaches
Phenotypic convergence	Independent genetic trajectories converging on similar resistant states	Cross-resistance across therapies; reproducible refractory phenotypes	Enables identification of shared functional vulnerabilities across patients
Systems-level outcome	Integration of genetic, epigenetic, metabolic, immune, and microenvironmental layers	RRMM behaves as a dynamic adaptive system rather than a static clonal disease	Supports adaptive, state-aware therapeutic strategies

## Data Availability

Not applicable. No new data were created or analyzed in this study.

## Abbreviations

The following abbreviations are used in this manuscript:

AML	Acute Myeloid Leukemia
BCMA	B-cell Maturation Antigen
BM	Bone Marrow
BsAb(s)	Bispecific Antibody(ies)
CAR-T	Chimeric Antigen Receptor T cells
CART	Chimeric Antigen Receptor T cells
CSC	Cancer Stem Cell
CXCR4	C-X-C Motif Chemokine Receptor 4
DNA	Deoxyribonucleic Acid
EMT	Epithelial–Mesenchymal Transition
FDG-PET	Fluorodeoxyglucose Positron Emission Tomography
GPRC5D	G Protein-Coupled Receptor Class C Group 5 Member D
HSP90	Heat Shock Protein 90
IFN	Interferon
IGF-1	Insulin-Like Growth Factor-1
IL-6	Interleukin-6
IMiDs	Immunomodulatory Drugs
MAPK	Mitogen-Activated Protein Kinase
mAb(s)	Monoclonal Antibody(ies)
MGUS	Monoclonal Gammopathy of Undetermined Significance
MM	Multiple Myeloma
MRD	Minimal Residual Disease
NCI	National Cancer Institute
NF-κB	Nuclear Factor kappa-light-chain-enhancer of activated B cells
NK cells	Natural Killer cells
PD-1	Programmed Cell Death-1
PD-L1	Programmed Death-Ligand 1
PI(s)	Proteasome Inhibitor(s)
RNA	Ribonucleic Acid
ROS	Reactive Oxygen Species
RRMM	Relapsed and Refractory Multiple Myeloma
SDF-1 (CXCL12)	Stromal Cell-Derived Factor-1
STAT	Signal Transducer and Activator of Transcription
TME	Tumor Microenvironment
UPR	Unfolded Protein Response
